# Toxicity of internalized polyalanine to cells depends on aggregation

**DOI:** 10.1038/s41598-021-02889-6

**Published:** 2021-12-06

**Authors:** Yutaro Iizuka, Ryuji Owada, Takayasu Kawasaki, Fumio Hayashi, Masashi Sonoyama, Kazuhiro Nakamura

**Affiliations:** 1 Department of Laboratory Sciences, Gunma University Graduate School of Health Sciences, 3-39-22, Showa-machi, Maebashi, Gunma 371-8511 Japan; 2 Accelerator Laboratory, High Energy Accelerator Research Organization, 1-1 Oho, Tsukuba, Ibaraki 305-0801 Japan; 3 Center for Instrumental Analysis, Organization for Promotion of Research and University Industry Collaboration, Gunma University, 1-5-1, Tenjin-cho, Kiryu, Gunma 376-8515 Japan; 4 Division of Molecular Science, Faculty of Science and Technology, Gunma University, 1-5-1 Tenjin-cho, Kiryu, Gunma 376-8515 Japan; 5 Gunma University Initiative for Advanced Research (GIAR), Gunma University, 1-5-1 Tenjin-cho, Kiryu, Gunma 376-8515 Japan; 6 Gunma University Center for Food Science and Wellness (GUCFW), Gunma University, 1-5-1 Tenjin-cho, Kiryu, Gunma 376-8515 Japan

**Keywords:** Neuroscience, Medical research, Chemistry

## Abstract

In polyalanine (PA) diseases, the disease-causing transcription factors contain an expansion of alanine repeats. While aggregated proteins that are responsible for the pathogenesis of neurodegenerative disorders show cell-to-cell propagation and thereby exert toxic effects on the recipient cells, whether this is also the case with expanded PA has not been studied. It is also not known whether the internalized PA is toxic to recipient cells based on the degree of aggregation. In this study, we therefore prepared different degrees of aggregation of a peptide having 13 alanine repeats without flanking sequences of PA disease-causative proteins (13A). The aggregated 13A was spontaneously taken up by neuron-like cultured cells. Functionally, strong aggregates but not weak aggregates displayed a deficit in neuron-like differentiation in vitro. Moreover, the injection of strong but not weak 13A aggregates into the ventricle of mice during the neonatal stage led to enhanced spontaneous motor activity later in life. Thus, PA in the extracellular space has the potential to enter adjacent cells, and may exert toxicity depending on the degree of aggregation.

## Introduction

Trinucleotide repeats are a class of microsatellites that can be found in a significant number of genes. The repeats include CAG, CGG, CCG, CTG, CUG, GAA and GCN^1^. The expansion of these repeats in certain genes produces proteins with expanded amino acid repeats. These proteins tend to form unusual structures and are toxic to the cells. Multiple mechanisms such as impaired axonal transport, increased autophagy and mitochondrial dysfunction have been suggested to underlie the toxicity of aggregated proteins^2^. As a result, the expansion often leads to pathological states, including neurological and developmental disorders^3^. These neurological disorders are usually progressive (e.g., polyglutamine [polyQ] diseases), and the number of repeats determines the severity and time of disease onset^1^. Intriguingly, polyQ-expanded Huntingtin, the causative protein for Huntington’s disease, aggregates and can be transferred between cells^4^, This phenomenon has also been seen in other misfolded proteins responsible for neurodegenerative disorders^5^.

Polyalanine (PA) diseases are another class of triplet expansion diseases. PA expansions are often observed in multiple transcription factors that play crucial roles in development; thus, most PA diseases are congenital. These transcription factors, namely, PHOX2B, RUNX2, HOXA13, SOX3, FOXL2, ZIC2, ARX and HOXD13 are associated with the following disorders: congenital central hypoventilation syndrome, cleidocranial dysplasia, hand-foot-genital syndrome, hypopituitarism, blepharophimosis, ptosis epicanthus inversus syndrome, holoprosencephaly, Partington syndrome and synpolydactyly type II, respectively. Additionally, oculopharyngeal muscular dystrophy, which is a late-onset PA disease, is caused by PABPN1. PABPN1-containing nuclear aggregates have been found in patients with oculopharyngeal muscular dystrophy, and intriguingly, other proteins, such as Hsp70 and HnRNP, co-localized with the aggregates^6,7^. However, whether the aggregated PA can be transferred between cells has not been explored.

Both the loss of normal function and a toxic gain-of-function can be generally observed in hereditary genetic diseases. Studies using genetically engineered mice with expanded alanine in PA disease-causative proteins showed a reduction in the levels of the protein^8^. The phenotypes observed in the mice with longer PA repeats were shared by other mutant mice with reduced levels of the same protein^9^. This could indicate that PA expansion may contribute to the loss-of-normal function of PA disease-causative proteins.

In terms of toxic gain-of-function, the transgenic expression of PABPN1 with expanded alanine induces intranuclear aggregation and apoptosis in skeletal muscle fibers^10–13^. Therefore, if PA aggregates are transferred from cell to cell, whether exogenous PA is toxic to the recipient cells may depend on the degree of aggregation.

In this study, we showed that an aggregated peptide with 13 alanine repeats without flanking sequences of PA disease-causative proteins can enter cells. Using neuron-like cells with different degrees of aggregated 13A, we studied whether the degree of aggregation affects neural differentiation in culture. Furthermore, we injected the 13A peptides into the brains of neonatal mice and conducted behavioral analyses later in life.

## Results

### Formation of solid aggregates from a peptide with 13A after incubation at 37 °C

To examine whether incorporated PA is toxic to cells, PA peptides had to first be aggregated. In addition, different degrees of aggregated PA needed to be prepared to test whether the toxicity depended on the degree of aggregation. A previous study tested the degree of aggregation of PA peptides using various lengths of alanine repeats under electron microscopy^14^, and the peptide with 13 alanine repeats (13A) was shown to have a moderate level of structure complexity. Therefore, we chose the peptide containing 13A. The sequence of 13A (KKWA_13_KK) was also determined based on this previous study^14^. In order to detect of the aggregates under a confocal microscope, 13A was fluorescently labeled with TAMRA.

**Figure 1:**
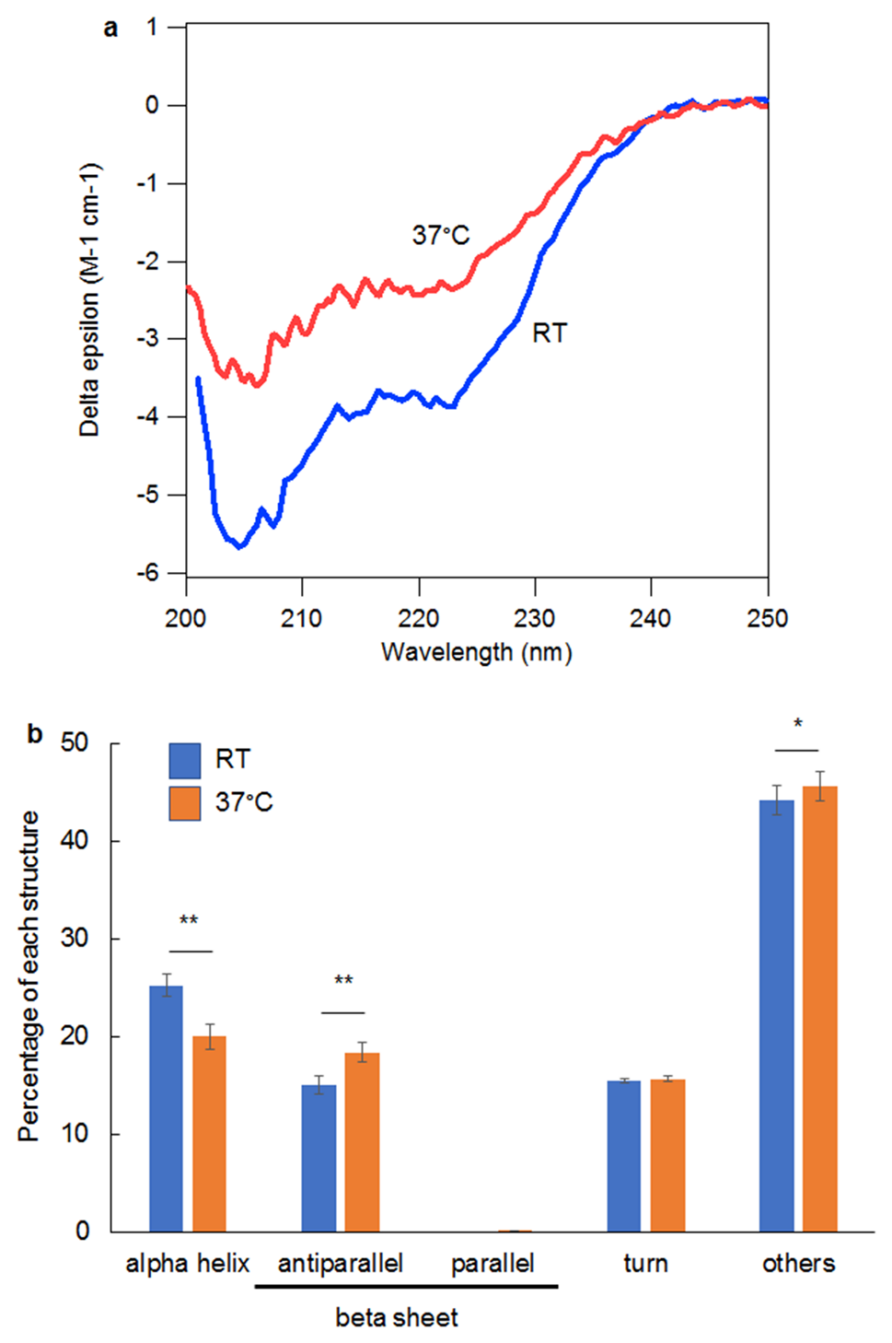
Secondary structure of a peptide with 13 alanine repeats under multiple temperature conditions. (**a**) Representative circular dichroism spectra of a 13A peptide (100 µg/mL in water) incubated for 4 h at room temperature (RT) or 37 °C. (**b**) The spectra (each n = 18) were analyzed using the BeStSel software to determine the percentages of following: alpha-helix, anti-parallel beta-sheet, parallel beta-sheet, turn and other structures. Each error bar represents the SE. ANOVA followed by posthoc test, *p < 0.05, **p < 0.01.

Aggregation of peptides partly depends on their secondary structure. Thus, we initially determined the secondary structure of 13A using circular dichroism (CD) analysis. Generally, longer incubation times and higher temperatures lead to stronger aggregation. We first induced aggregation of 13A at 37 °C for 7 d at a concentration of 1 mg/mL in water. The aggregated 13A was further diluted to 100 µg/mL in water for the CD analysis. The far-UV CD spectra were obtained from 13A peptides over a wavelength range of 200–250 nm at room temperature (RT). Since the 13A peptides were labeled with the fluorescent dye TAMRA, we also obtained CD spectra from TAMRA alone and subtracted them from those of the TAMRA-labeled 13A peptides. However, the sample showed very low CD levels over the wavelength range of 200–250 nm. Therefore, we decided to apply a fixed shorter incubation time (4 h) and compare the CD value between the 13A incubated at RT and that incubated at 37 °C. Under these conditions, both samples had significant CD values (Fig. 1a). The data were analyzed using BeStSel software to quantify the percentages of secondary structures. This software can be used to individually quantify parallel and anti-parallel beta-sheets. The 13A contained an anti-parallel beta-sheet, likely due to the amino acid sequence. The content of parallel beta-sheet was approximately 0%. The percentage of alpha-helix was lower in the 13A incubated at 37 °C than at RT (Fig. 1b). Conversely, the contents of the anti-parallel beta sheet and others (e.g., random coil structure) were higher at 37 °C than at RT (Fig. 1b). While beta-sheet structure is related to amyloid formation^15,16^, alpha-helix and random coil are not necessarily related. These results suggest that the incubation of 13A at 37 °C rather than at RT might result in stronger aggregates.

**Figure 2:**
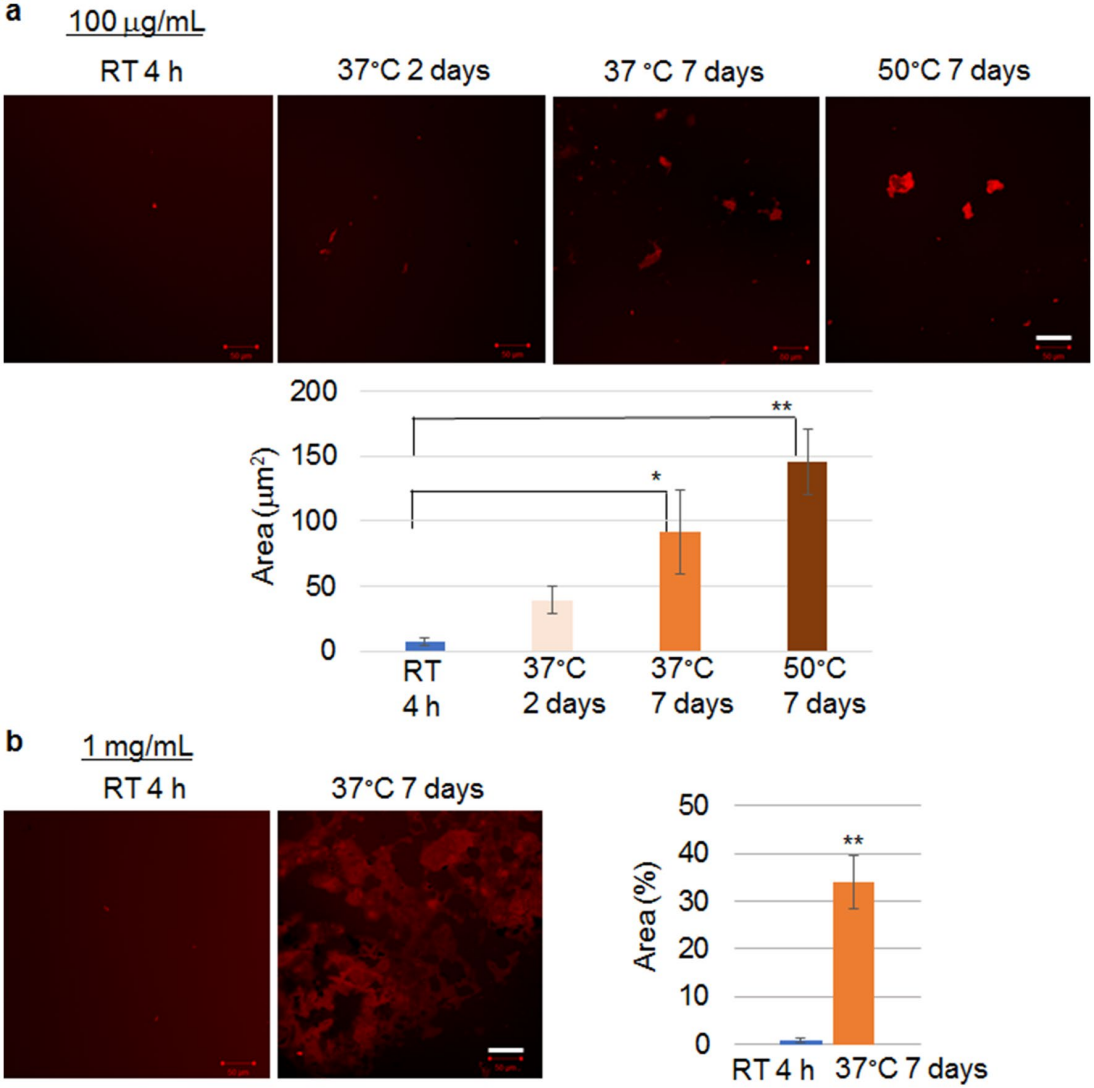
Comparison of the aggregation of peptides with 13 alanine repeats under multiple concentrations, temperatures, and incubation times. (**a**) Quantification of the area of aggregate clusters of TAMRA-labeled 13A peptide (100 µg/mL, red) incubated for 4 h at room temperature (RT) (n = 15 from 7 images), for 2 d at 37 °C (n = 15 from 9 images), for 7 d at 37 °C (n = 14 from 7 images), or for 7 d at 50 °C (n = 16 from 6 images) (bottom). Representative confocal images are shown (top). ANOVA followed by posthoc test, *p < 0.05, **p < 0.01. (**b**) TAMRA-labeled 13A peptide (1 mg/mL, red) incubated for 4 h at RT or for 7 d at 37 °C. Representative confocal images are shown (left). Quantification of the percentage of the area occupied by aggregates in a field of view (n = 5 each) (right). Each error bar represents the SE. Student’s t-test, **p < 0.01. Scale bars, 50 µm.

The differential pattern of the secondary structure between the two temperatures motivated us to compare the size of clusters of 13A aggregates under multiple temperatures and incubation times. We initially induced aggregation at a concentration of 100 µg/mL in PBS because in our previous study, a concentration of 100 µg/mL in aqueous solution produced a solid aggregation of polyQ peptides^17^. Confocal images showed the formation of small clusters of aggregates after incubation with 13A for 4 h at RT (Fig. 2a). Notably, the 13A that was incubated for 7 d at 37 °C appeared to form larger clusters than those incubated for 4 h at RT (Fig. 2a). We then observed cluster formations at temperatures > 37 °C. The size of the clusters after incubation for 7 d at 50 °C was not significantly different from that after incubation for 7 d at 37 °C (Fig. 2a). The size of the clusters at 1 mg/mL was then compared between those incubated for 4 h at RT and for 7 d at 37 °C. Higher concentrations led to larger clusters (Fig. 2b). When we quantified the percentage of area occupied by the aggregates in the field of view, the percentage was much higher in the aggregates prepared by incubation for 7 d at 37 °C than for 4 h at RT (Fig. 2b).

**Figure 3:**
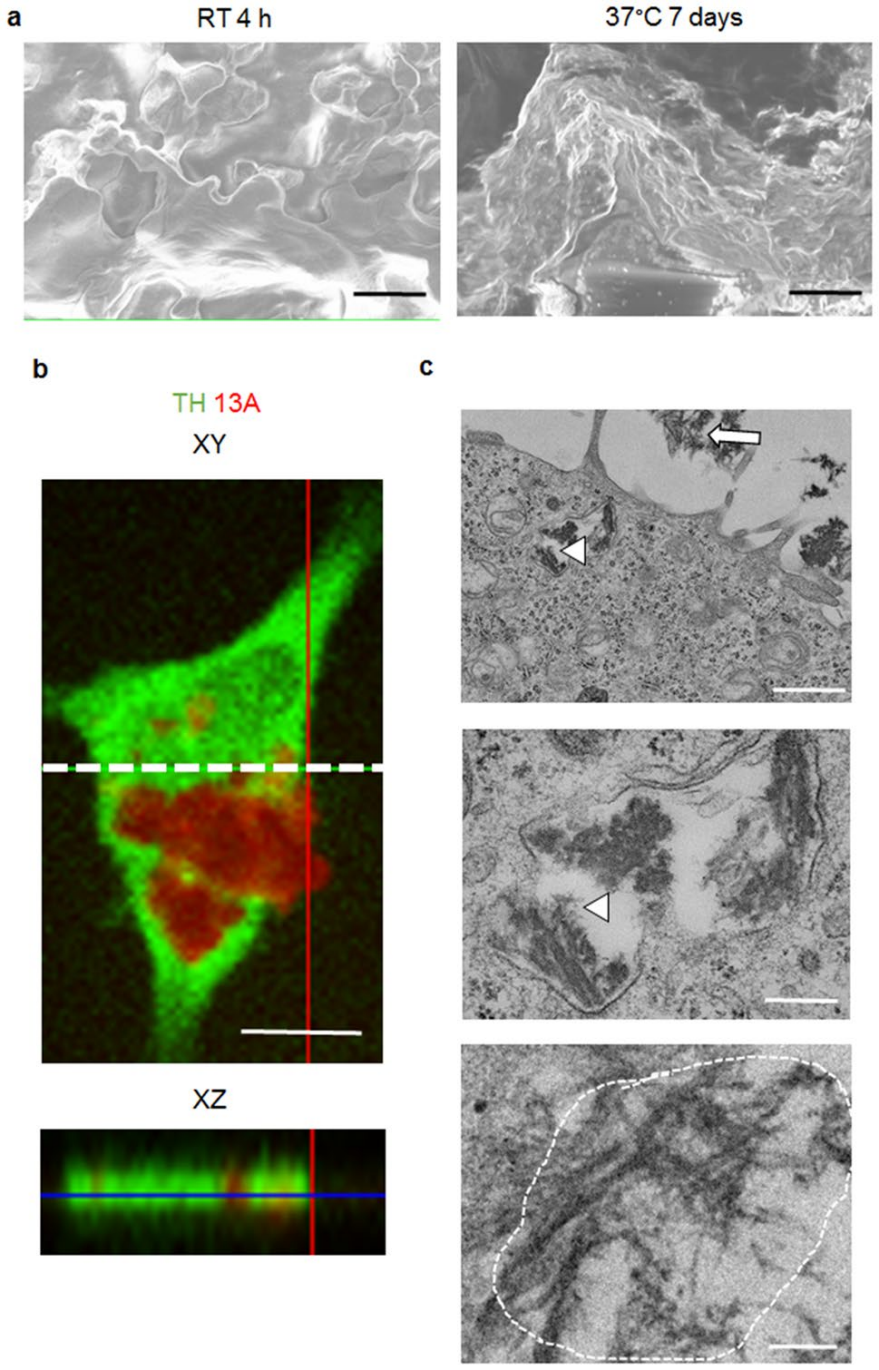
Aggregated 13A is taken up by PC12 cells. (**a**) Scanning electron microscopy images of a TAMRA-labeled 13A peptide (1 mg/mL) incubated for 4 h at RT or 37 °C for 7 d. Each representative image was chosen from 5 images. Scale bars, 5 µm. (**b**) An ortho XZ-axis cross section confocal image of a PC12 cell (bottom) along the dotted line in the XY-axis view (top) was reconstituted from z-stacks using confocal laser scanning microscopy. The interval of z-stacks was set at 1 µm. The cell stained with TH antibody (green) has 13A signals (red). Scale bar, 10 µm. (**c**) Transmission electron microscopy images of PC12 cells with an uptake of aggregated 13A. TAMRA-labeled 13A peptides (1 mg/mL) incubated at 37 °C for 7 d were added to PC12 cells to a concentration of 10 µg/mL in the culture medium. The cells were then cultured for one day and subjected to transmission electron microscopy. The arrow and arrowheads indicate 13A aggregates outside and inside the cell, respectively (top, middle). The 13A aggregates inside the cell are magnified (middle). The fibril-like structure is surrounded by dotted line (bottom). The representative images were chosen from 58 images. Scale bars, 5 μm (top), 1 µm (middle, bottom).

Furthermore, the aggregates prepared by incubation for 7 d at 37 °C, but not for 4 h at RT, were fibrous on scanning electron microscopy imaging (Fig. 3a). Collectively, for the following analyses, we decided to incubate for 4 h at RT and 7 d at 37 °C to produce weak and strong aggregates, respectively.

### Highly-aggregated 13A alters cellular functions in vitro

The vast majority of expanded PA-containing proteins play multiple roles in a variety of cell types during normal development. Since a previous study of mutant mice with an expanded repeat of the codon GCG in the Arx gene displayed disorganized architecture in the neocortical layers and hippocampus and exhibited spasms in infancy^18^, we attempted to examine whether incorporated 13A leads to toxicity in neural development in vitro. In addition, we compared strong and weak aggregates to determine whether the toxicity was dependent on the degree of aggregation.

To mimic neural development in culture, we used PC12 cells, which can be differentiated into neuron-like cells after stimulation with NGF. For functional analysis, aggregated 13A peptides need to be taken up by PC12 cells, as seen in aggregated polyQ^17,19^. To determine whether 13A aggregates are taken up by PC12 cells, we first induced aggregation at 1 mg/mL and added the aggregates to the culture media of PC12 cells to a final concentration of 10 µg/ml. We then took z-stack confocal images at every 1 µm and obtained the ortho image along the vertical axis (Fig. 3b). The fluorescent signals of the strong aggregates were found between cytoplasmic tyrosine hydroxylase (TH) signals, indicating that 13A was located inside the PC12 cells. Furthermore, transmission electron microscopy images were obtained using the PC12 cells to confirm the above result. Many aggregates were observed outside the cells (Fig. 3c, top). Parts of the aggregates showed fibril-like structures (Fig. 3c, middle, bottom). Fibrils are elongated, insoluble protein aggregates deposited in vivo in amyloid diseases^20^. Notably, the aggregates inside the cells showed same appearance as those outside the cells with same electron density (Fig. 3c). These results indicate that the aggregates were taken up by PC12 cells.

**Figure 4:**
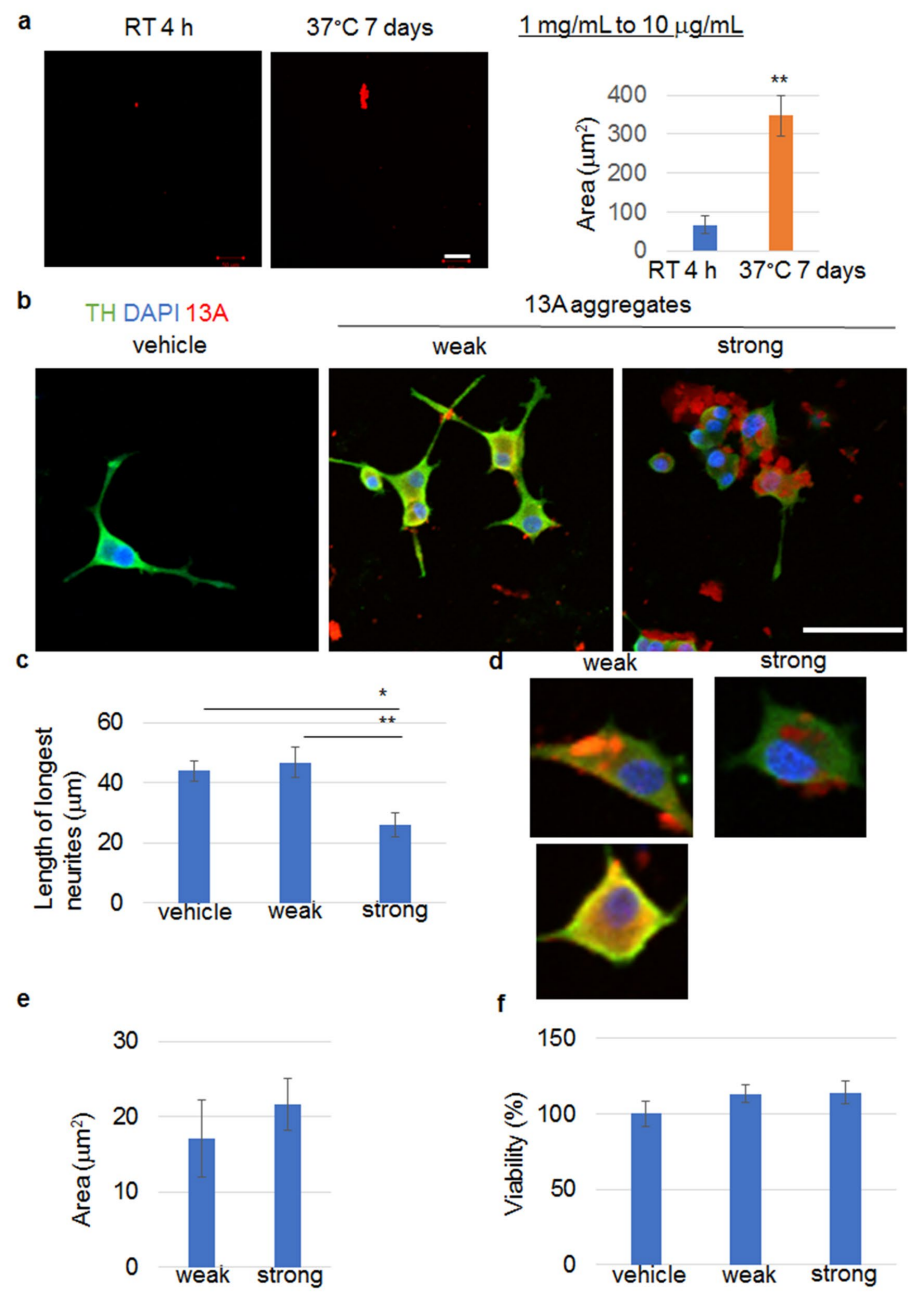
Incorporation of 13A with strong aggregation disturbs the differentiation of PC12 cells. (**a**) TAMRA-labeled 13A peptides (1 mg/mL, red) incubated for 4 h at RT or for 7 d at 37 °C were added to the culture medium to a final concentration of 10 µg/mL and were then further incubated for 5 d at 37 °C. Representative confocal images are shown (left). Quantification of the area of aggregate clusters (n = 10 from 9 images and n = 14 from 11 images for 4 h at RT and 7 d at 37 °C, respectively) (right). Each error bar represents the SE. Student’s t-test, **p < 0.01. (**b**–**e**) 13A peptides were incubated at RT or at 37 °C before being added to the PC12 cells. The resultant weak and strong 13A aggregates were added to the culture media and the aggregates (red) were taken up by the PC12 cells. Vehicle-treated PC12 cells were also prepared. The PC12 cells for the 3 groups were cultured for 5 d after NGF was used to induce differentiation. The cells were then stained with TH antibody (green) and DAPI (blue). Representative images are shown (**b**). Length of longest neurites was quantified (n = 35 from 13 images, 49 from 19 images and 31 from 14 images for vehicle, weak aggregates and strong aggregates, respectively) (**c**). The area of the aggregate clusters were quantified per cell (n = 25 and n = 23 for weak and strong aggregates, respectively) (**d**,**e**). (**f**) The viability of undifferentiated PC12 cells were examined 1 day after adding the vehicle, weak aggregates, or strong aggregates using Cell Counting Kit-8. The values relative to that of vehicle-treated cells were used. Each error bar represents SE. ANOVA, *p < 0.05, **p < 0.01. Scale bars, 50 µm.

For functional analysis, PC12 cells were maintained at 37 °C for several days. Even for the weak aggregates formed after incubation for 4 h at RT, the clusters of aggregates could become larger after several days of culture at 37 °C and eventually be identical to those of the strong aggregates (incubated initially at 37 °C for 7 d) added to cultured cells. To test this possibility, we added the aggregates that were incubated for 4 h at RT and those incubated for 7 d at 37 °C at a concentration of 1 mg/mL to culture media in 24-well culture plates to a final concentration of 10 µg/ml in the media. After 5 d in a CO2 incubator at 37 °C, the area of aggregate clusters formed by those incubated for 4 h at RT was still significantly smaller than those incubated at 37 °C for 7 d (Fig. 4a). Since the aggregates attach to the bottom of the culture plate a short time after being added to the medium, the difference is likely to be maintained after incubation at 37 °C.

We then carried out functional analyses using the PC12 cells treated with the vehicle, weak aggregates (RT for 4 h), or strong aggregates (37 °C for 7 d). PC12 cells from the three groups were cultured for 5 d after the induction of neuron-like differentiation with NGF. The cells with neurites that were longer than the size of the cell body were defined as the differentiated cells. Upon induction, 22% of the vehicle-treated cells differentiated. Those treated with weak aggregates had the same degree of differentiation (24%), while those treated with strong aggregates had a low degree of differentiation (10%). To assess neuritic outgrowth, the lengths of the longest neurites were measured. The length of the neurites on the cells treated with the weak aggregates was not significantly different from that on the vehicle-treated cells (Fig. 4b,c). However, the length was significantly shorter on the cells treated with strong aggregates compared to the other two groups (Fig. 4b,c). Thus, both NGF-induced differentiation and neurite extension were impaired in cells with strong aggregates but not in cells with weak aggregates.

In some cells treated with weak aggregates, 13A appeared as the focal cluster of aggregates in the cells (top of Fig. 4d). However, in other cells, the 13A showed a uniform distribution (bottom of Fig. 4d). In contrast, the 13A in the cells treated with strong aggregates showed a focal cluster pattern (Fig. 4d). The total area of the aggregates per cell was not significantly different between the PC12 cells with weak aggregates and those with strong aggregates (Fig. 4e). Moreover, a larger area of aggregates did not appear to lead to more severe morphological deficits, because the area of aggregates and length of the longest neurite were not correlated (correlation coefficient, 0.09; p = 0.62). Collectively, this morphological difference was likely not due to the amount of aggregates , but rather to differences in the secondary structure and ultrastructure of the aggregates.

A cell viability assay was then performed using a formazan dye to measure the activities of dehydrogenases in undifferentiated PC12 cells 1 d after the addition of aggregates. Viability was not different among the vehicle, weak aggregate, or strong aggregate groups (Fig. 4f).

### Enhanced spontaneous motor activity in mice given highly-aggregated 13A

**Figure 5:**
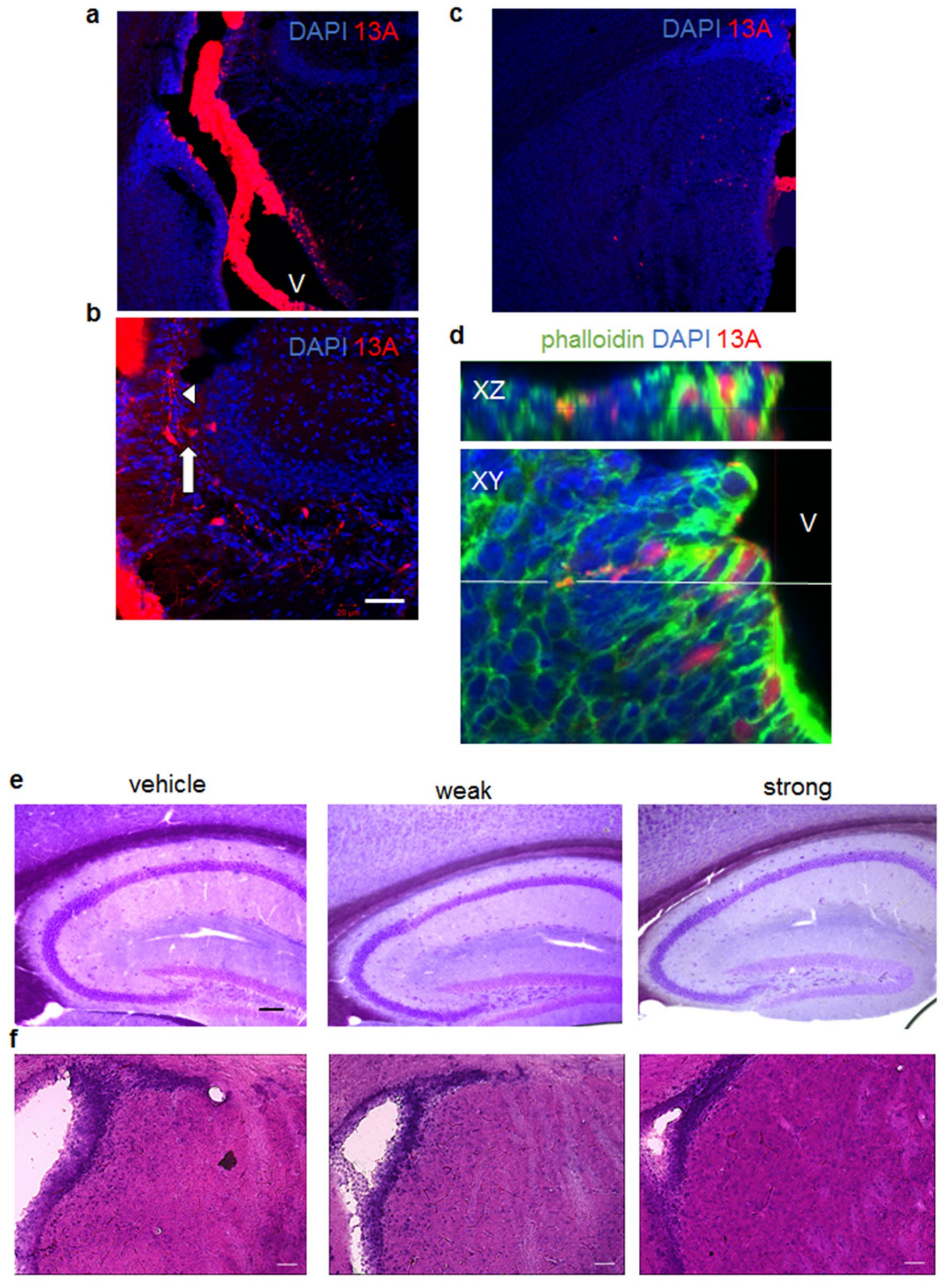
No gloss anatomical changes in mice given 13A at P2. (**a**–**d**) Fluorescence images of coronal brain sections of mice at postnatal day 2 (P2) one hour after TAMRA-conjugated 13A peptide with strong aggregation (red) was injected into the ventricle. The sections were stained with phalloidin (green) and/or DAPI (blue). The 13A signals are found in the ventricle (V) and also in the brain parenchyma that are close to the ventricle (**a**). The 13A signals in the hippocampus (**b**) and the striatum (**c**) are shown. An ortho XZ-axis cross section confocal image of the brain (top) along the white line on XY-axis view (bottom) was reconstituted from the z-stacks using confocal laser scanning microscopy (**d**). The interval of z-stacks was set at 1 µm. The arrow and arrowhead in (**b**) indicates13A-positive cell bodies and processes, respectively. Scale bar, 50 µm. (**e**) Nissl-stained sagittal sections of the hippocampus from 1-month-old mice treated with vehicle, weak aggregates and strong aggregates. Each representative image was chosen from 14 images. Scale bar, 100 µm. (**f**) HE staining of coronal sections of the striatum from 1-month-old mice treated with vehicle, weak aggregates and strong aggregates. Representative images were chosen from 14, 7, 11 images for vehicle, weak aggregates and strong aggregates, respectively. Scale bars, 50 µm.

We then sought to determine whether exogenously applied aggregated 13A alters brain functions in vivo. To this end, we examined whether 13A injected into the lateral ventricles at postnatal day 2 (P2) is found in the brain parenchyma. TAMRA-conjugated strongly-aggregated 13A were found in the ventricle 1 h after the injection and also in the brain parenchyma that were close to the ventricle (Fig. 5a). We found many 13A signals in the hippocampus (Fig. 5b) and the striatum (Fig. 5c), although fewer 13A signals were also detected in other regions. The 13A signals were detected in the cell body, some of which were also located in the cell processes (Fig. 5b). Confocal ortho imaging was used to verify the localization of 13A between the phalloidin signals (Fig. 5d), suggesting that parts of the 13A signals are located inside the cells.

We checked whether 13A induces morphological disorganization in the hippocampus and the striatum. In line with the normal viability of PC12 cells treated with 13A (Fig. 4f), Nissl staining of mice at 1 month of age revealed that no gloss anatomical changes had occurred in the hippocampus (Fig. 5e). Similarly, 13A did not induce gloss morphological changes in the wall of ventricle and the striatum, as evidenced by HE staining (Fig. 5f).

**Figure 6:**
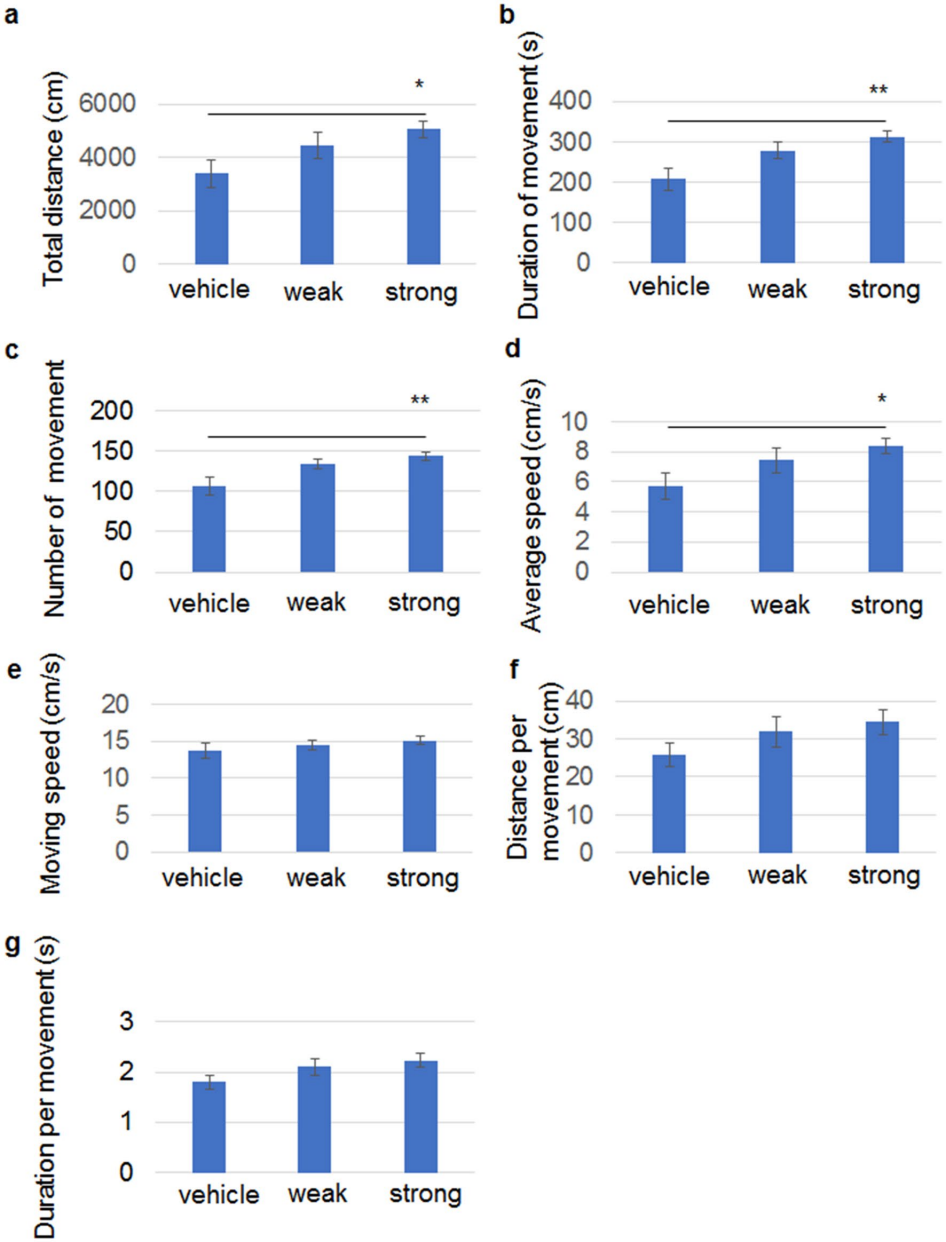
Strong aggregates of 13A increases spontaneous motor activities at 2 weeks of age. An open field test was conducted using 2-week-old mice that received an injection of the vehicle, weak aggregates, or strong aggregates at P2 (n = 14, each). The indices used were total walking distance (**a**), duration of movement (**b**), number of movement episode (**c**), average speed of locomotion over 10 min (**d**), moving speed (**e**), distance per movement (**f**) and duration per movement (**g**). Each error bar represents SE. ANOVA followed by post hoc test, *p < 0.05, **p < 0.01.

Since the hippocampus and the striatum are pivotal regions to regulate and control locomotion^21–23^, spontaneous motor activity was studied in an open field using 2-week-old mice that received an injection of 13A at P2. The total walking distance (Fig. 6a) and duration of movement (Fig. 6b) were longer in the mice given strong aggregates than in vehicle-treated mice. Similarly, the number of movement episodes was higher (Fig. 6c), and the average speed of locomotion over 10 min was faster (Fig. 6d) in the mice given strong aggregates. Speed during movement (Fig. 6e), distance per movement (Fig. 6f), and duration per movement (Fig. 6g) were not different between the groups. None of the parameters were significantly different between vehicle-treated mice and weak aggregate-treated mice. Collectively, spontaneous motor activity was elevated in the mice treated with strong aggregates at 2 weeks of age.

**Figure 7:**
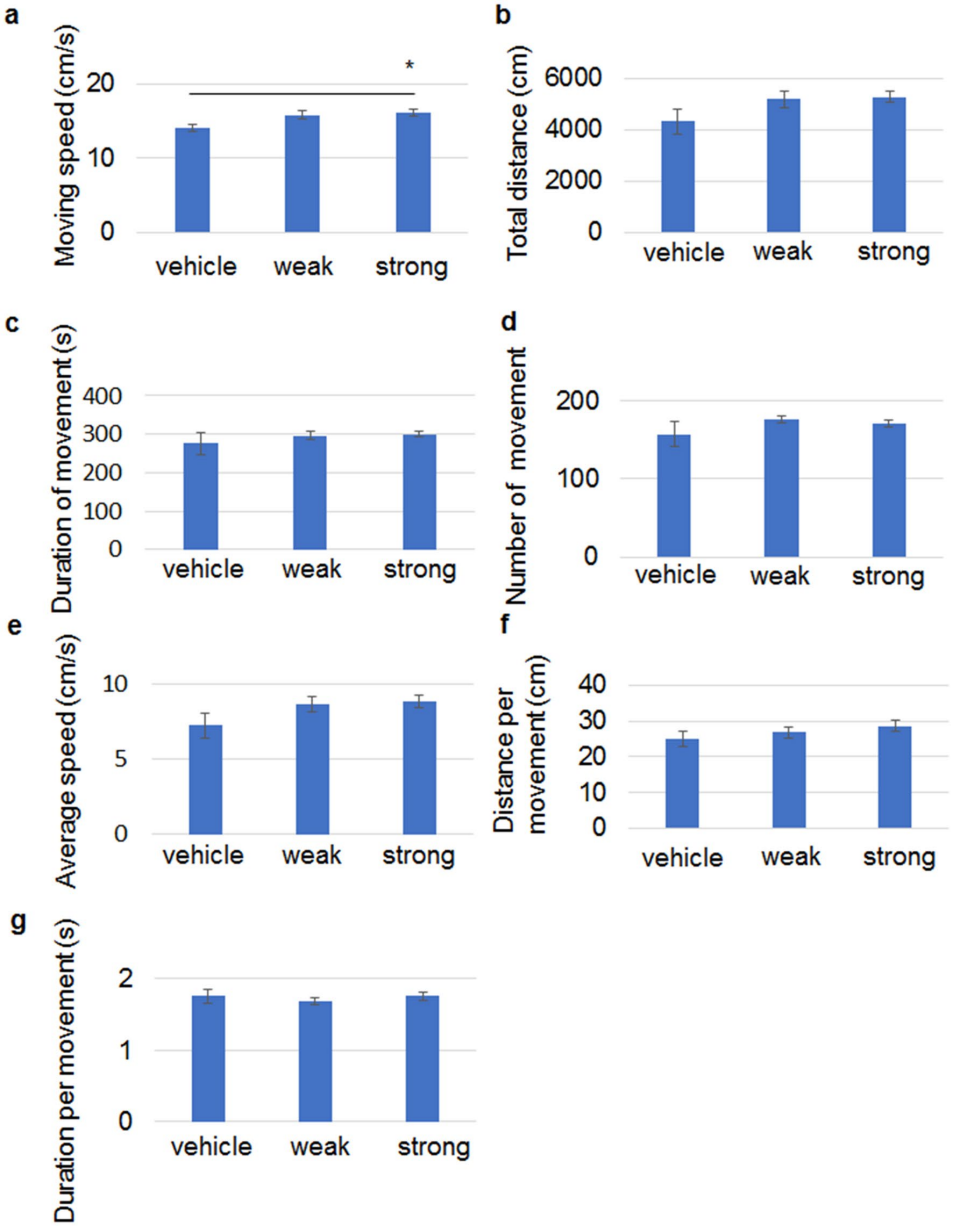
Strong aggregates of 13A increases moving speed in open field at 1 months of age. Open field test of 1-month-old mice that received an injection of vehicle (n = 11), 13A with weak aggregation (n = 17) or 13A with strong aggregation (n = 13) at P2. The indices used were moving speed (**a**), total walking distance (**b**), duration of movement (**c**), number of movement episode (**d**), average speed of locomotion over 10 min (**e**), distance per movement (**f**) and duration per movement (**g**). Each error bar represents SE. ANOVA followed by post hoc test, *p < 0.05.

At 1 month of age, only moving speed was faster in the mice treated with strong aggregates compared to vehicle-treated mice (Fig. 7a), while the other parameters were not different (Fig. 7b–g). Weakly-aggregated 13A did not change the moving speed.

## Discussion

Almost 50% of the diseases caused by triplet repeats in genes are polyQ diseases^24^. Huntington’s disease, spinocerebellar ataxia type 1 (SCA1), SCA2, SCA3, SCA6, SCA7, SCA17, spinal and bulbar muscular atrophy, and dentatorubropallidoluysian atrophy are classified as polyQ diseases^24^. However, the fact that polyleucine encoded with mixed DNA repeats has been found to be significantly more toxic than polyQ in mammalian cells^25^ justifies the exploration of the pathogenesis of other triplet expansion diseases, such as PA diseases.

The molecules that are the causative agents of neurodegenerative disorders are characterized by a conversion from a folded to a misfolded structure. A general property shared by these proteins is cell-to-cell transmission^4^. Among the proteins responsible for polyQ diseases, mutant Huntingtin aggregates can move between individual co-cultured cells. Even in a Drosophila model, mutant Huntingtin aggregates were found to transfer from presynaptic neuronal axons to phagocytic glia^26^. The transmission process includes both the release of aggregates from the cell and the uptake of the aggregates by another cell. Since we added 13A aggregates to the culture medium of PC12 cells, we studied the uptake of 13A aggregates. Transmission electron microscopy revealed that the 13A aggregates were extensively taken up by PC12 cells, similar to what has been seen with polyQ aggregates^19^. Our observation suggests that, in vivo, aggregated PA derived from dead cells in the extracellular space may be taken up by adjacent cells, where the aggregates have adverse effects on the recipient cells. Thus, it is worth studying whether the proteins that contain expanded PA and are responsible for PA diseases are also capable of entering cells.

The uptake of Huntingtin exon 1 aggregates has been found to be reduced by the application of inhibitors for clathrin-dependent endocytosis using a neuroblastoma cell line^27^. The possible involvement of endocytosis in the uptake of aggregated proteins that are responsible for other neurodegenerative disorders has also been reported^28–30^. Therefore, endocytosis may also be implicated in the entry of PA. Indeed, electron microscopy images have revealed that 13A aggregates inside the cells are surrounded by a cell membrane-like bilayer structure, with empty spaces between the aggregates and cell membrane-like structures. Thus, endocytosis might be the mechanism by which 13A aggregates are taken up by cells. Future studies using endocytosis inhibitors are necessary to confirm this point.

Moreover, internalized polyQ aggregates in recipient cells have the prion-like capacity of transference, by which the internalized aggregates nucleate the aggregation of normal polyQ-containing proteins^4^. Accordingly, the initial focus of polyQ diseases in the brain spreads from more susceptible regions to less susceptible regions. We hope to test this point in the near future using aggregated PA.

Although the pathogenesis of PA diseases has not yet been thoroughly investigated, accumulating evidence regarding the pathogenesis of polyQ diseases^31–37^ provides clues for predicting the mechanisms of PA diseases. Of particular interest is the question of whether PA aggregation itself contributes to toxicity because the propensity of aggregation was proposed to be an accurate surrogate for polyQ toxicity rather than the repeat length of polyQ itself using an engineered analog representing Huntingtin exon1^38^. Several studies have linked PABPN1 aggregates to cell death^10–13^. Similarly, aggregates consisting of HOXD13 with expanded PA repeats prevent normal HOXD13 from entering the nucleus^39^. However, since different lengths of PA exhibit different degrees of aggregation, we cannot distinguish the effect of a longer length of PA from that of a higher degree of aggregation when expanded PA is overexpressed or is knocked-in. One strategy to distinguish between these two is to use the same PA-containing protein with different degrees of aggregation. In this study, we addressed this point by introducing the same PA peptides to the cells with differential degrees of aggregation.

Using PC12 cells containing 13A, we found that strong 13A aggregates disturbed neurite outgrowth after NGF stimulation, while weak aggregates did not cause any impairment. This result implies that the aggregation itself inhibits the development of neurons, at least in the case of 13 alanine repeats. In this study, we showed that strong aggregates have lower alpha-helix content and higher beta-sheet content than weak aggregates. In accordance with the secondary structural changes, a fibril-like structure appeared on parts of the strong aggregates, as confirmed by transmission electron microscopic analysis. Indeed, previous studies have revealed the presence of amyloid fibrils in PA^40,41^. Our previous studies have revealed that the specific wavelength of mid-infrared laser irradiation on polyQ aggregates increases the content of alpha-helix and decreases the content of beta-sheet^17^, which ameliorates the deficit in neurite outgrowth of neuron-like SH-SY5Y cells caused by polyQ^19^. Therefore, beta-sheet-related aggregation might disturb neural development, regardless of the type of protein. However, we cannot exclude the potential toxicity resulting from the alpha-helix of PA, since the alpha-helix was a dominant structure of EGFP-tagged PA with 37 alanine repeats^42^. Thus, the repeat length of alanine likely affects the composition of the secondary structure. Moreover, we should note that the results above were not derived from primary neurons but rather from neuron-like cells. We are planning to test the effects of 13A in primary neurons in the future, providing that we are able to find suitable conditions to maintain primary neurons with 13A.

We also tested the effects of the aggregation on spontaneous motor activity at both 2 weeks and 1 month old and found an increased spontaneous motor activity in mice with the strong aggregates but not with weak aggregates. The injected aggregates were easily found in the hippocampus and the striatum. The initiation and/or execution of motor tasks are impaired in Huntington’s disease, and loss of inputs to the striatum from cortex and thalamus and also loss of outputs to the external pallidal segment were seen in patients with Huntington’s disease^43^. Moving speed was faster in mice with strong aggregates at 1 month old. Notably, the hippocampus controls the speed of locomotion^22,23^. At birth, neural circuits are not fully developed, as the mouse brain fully matures at around 3 weeks of age. Any deficits that arise before the development of the neural network confer irreversible brain dysfunction in adults. Although we did not detect gloss anatomical changes in the hippocampus and the striatum of 13A-injected mice, 13A in these regions might lead to functional deficits in control and modulation of motor activity. However, we cannot exclude the effect of 13A in other brain regions such as the cerebral cortex and cerebellum that control and modulate motor activity. Collectively, our results suggest that the toxicity of internalized 13A to recipient cells depends on the degree of aggregation. Since the PA peptide we used was 13A without flanking sequences of PA-causing molecules, toxicity elicited by aggregation might be a common feature for all expanded PA-containing proteins when they are internalized. However, whether the toxicity of endogenous expanded PA-containing proteins to cells is dependent on aggregation has not been determined.

Alternatively, some findings regarding PA suggest that loss-of-function is also involved, as has been shown with polyQ diseases^44^. This evidence was obtained through comparing phenotypes between animal models with reduced levels of PA disease-causative proteins and those with expanded PA in the same proteins. One study of heterozygous knock-in mice with expanded alanine in PABPN1 proteins showed changes in the poly A tails and the usage of poly A signals, mitochondrial damage, and myopathic phenotypes. Remarkably, the phenotypes were partially reproduced in heterozygous Pabpn1 knock-out mice^9^. Another study using RNA interference during brain development found impairments associated with the inactivation of Arx that resembled the microcephaly seen in the corresponding patients^45^. Using a functional rescue technique involving adding the corresponding protein, the loss-of-normal function of the protein was further verified. While the PA-expanded SOX3 protein could rescue a block in gastrulation seen in the deficient mice, it was not sufficient for normal brain development^8^. Intriguingly, adding normal PABPN1 resulted in the reestablishment of apoptosis in the PABPN1 with extended PA, although the number of nuclear aggregates increased^10^. In this case, the disadvantage of the loss of normal function was likely more detrimental than the toxicity of the aggregates. Thus, although the current observation generally justifies an attenuation of the degree of aggregation as a therapeutic approach for PA diseases, aggregation might not be a therapeutic target for some PA diseases. In addition, strong aggregation might not necessarily be more toxic than less-aggregated forms for some PA-causing proteins with expanded PA instead of PA alone, as interactome analyses have revealed more extensive detrimental interactions with polyQ soluble oligomers than with insoluble aggregates^46^. We hope to perform future studies to elucidate the pathogenic contribution of the aggregation of each expanded PA-containing protein.

## Methods

### Polyalanine peptides

Synthesized TAMRA-labeled peptides containing 13 uninterrupted alanine repeats (13A) had a purity of more than 95% (GL Biochem, Shanghai, China). The sequence is 5-TAMRA-KKWAAAAAAAAAAAAAKK-NH_2_. The flanking sequence of alanine repeat was determined according to a previous literature^14^. The stock solution of these peptides were made by dissolving the peptides in a 1:1 mixture of trifluoroacetic acid (TFA) and hexafluoroisopropanol (HFIP).

### Circular dichroism analysis

CD spectra were obtained using a J-820 spectropolarimeter equipped with a PTC-423L Peltier temperature controller (JASCO Co., Tokyo, Japan). The far-UV CD spectra were obtained from TAMRA-labeled 13A peptides (100 µg/mL in water) placed in a 0.1 cm cuvette at RT. The wavelength range of 200–250 nm scans was averaged for the CD spectra. The CD spectra were smoothed using the Savitzky-Golay algorithm. To eliminate non-specific values, TAMRA data without any peptides were subtracted from those of TAMRA-labeled 13A peptides. BeStSel software, which takes into account the twist of β-structures^47^, was applied to quantify the percentage of secondary structures.

### Scanning electron microscopy

The acceleration voltage of the FE-SEM Supra 40 scanning electron microscope (Zeiss, Oberkochen, Germany) was set at 5.0–10.0 kV to perform a detailed morphological analysis of the aggregated 13A peptides. The aggregated 13A peptide solution was dried on a glass slide, which was fixed on a sample holder using conductive copper tape.

### Cell culture

PC12 cells, which were purchased from RIKEN BRC, were cultured as previously described^19^. Briefly, 1 × 10^4^ PC12 cells were plated on a micro cover glass in 24-well plates. DMEM containing 10% FBS and antibiotics was used as the culture medium. After the addition of PA peptides at a concentration of 10 µg/ml, the PC12 cells were differentiated in DMEM containing 1% FBS, 0.25% BSA and 50 ng/mL NGF for 5 d in 5% CO_2_ at 37 °C for morphological analysis. NGF-induced differentiation was determined to have occurred if the cells had an extension of at least one neurite that was longer than the size of the cell body. Neurite outgrowth capacity was evaluated based on the length of the longest neurites. The viability of PC12 cells in 96-well plates was measured using the Cell Counting Kit-8 (Dojindo, Kumamoto, Japan) according to the manufacturer’s instructions. Absorbance at 450 nm was measured using a microplate reader.

### Transmission electron microscopy

The PC12 cells were fixed with 2% paraformaldehyde and 2% glutaraldehyde in a 0.1 M phosphate buffer (PB), pH 7.4, at 4 °C for 30 min. The cells were then fixed with 2% glutaraldehyde in 0.1 M PB at 4 °C overnight. After washing three times with 0.1 M PB for 30 min each, the cells were postfixed with 2% osmium tetroxide in 0.1 M PB at 4 °C for 1 h. After dehydration with graded ethanol, the cells were transferred to a resin (Quetol-812; Nisshin EM Co., Tokyo, Japan) and polymerized at 60 °C for 48 h. Ultra-thin sections of 70 nm thickness were prepared from the polymerized resins using an ultramicrotome (Ultracut UCT; Leica, Vienna, Austria) with a diamond knife. The sections mounted on copper grids were stained with 2% uranyl acetate at RT for 15 min. After washing with distilled water, the samples were stained again with a lead stain solution (Sigma) at RT for 3 min. The grids were observed using a transmission electron microscope (JEM-1400Plus, JEOL Ltd., Tokyo, Japan) at an acceleration voltage of 100 kV. Digital images of 3296 × 2472 pixels were captured using a CCD camera (EM-14830RUBY2, JEOL Ltd., Tokyo, Japan).

### Mice

The ICR mice underwent bilateral intracerebroventricular injections of PA solution (100 µg/mL in PBS, 1 µl) at P2, as described previously^48^. Briefly, the mice were restrained on ice before injection. A 10 µl Hamilton syringe needle was inserted at a site approximately 1.0 mm lateral to the sagittal suture, 1.0 mm caudal from the bregma, and 2 mm deep from the skull surface. Behavioral tests were performed at two and four weeks of age. The NIH guidelines for the treatment of mice were followed, and every effort was made to minimize the suffering of the mice and to reduce the number of mice used in the experiments. The animal study was performed in compliance with the ARRIVE guidelines, and the experiments were approved by the Animal Resource Committee of Gunma University. The mice were kept in a pathogen-free environment at a temperature of 23 °C, with a light/dark cycle of 12 h.

### Imaging

Staining was performed as previously described^19,49^. Briefly, PC12 cells were fixed with 4% paraformaldehyde and then incubated with anti-TH antibody (Merck Millipore, Burlington, MA). The secondary antibody used was cy2-labeled anti-rabbit IgG (Jackson ImmunoResearch, West Grove, PA, USA). For images of mouse brain sections, 50 µm thick coronal and sagittal brain sections were prepared using a freezing microtome after fixation with 4% paraformaldehyde and dehydration with 30% sucrose. The sections were used for HE staining, Nissl staining and fluorescence staining. For Nissl staining, the sections were stained with 0.1% cresyl-violet solution for 60 min at 37 °C. Fluorescence staining was carried out using Phalloidin-iFluor 488 conjugate (Cayman Chemical, Ann Arbor, MI) and DAPI. Images were taken using either an LSM 880 confocal microscope (Zeiss, Oberkochen, Germany) or a BZ9000 microscope (Keyence, Osaka, Japan).

### Quantification of peptide and cultured cells

The length of the neurites and the area of aggregates in PC12 cells were measured using Image J software as described previously^49^. Since 1.0 µm is the smallest length available for detecting differences using this software, processes greater than 1.0 µm were selected. For peptide aggregates, the area of the aggregates was also measured using Image J software.

### Open field test

All spontaneous movement was automatically measured in an open field (50 × 50 × 50 cm, O’HARA & CO., LTD., Tokyo, Japan) for 10 min. The mouse was first placed in the center of the field and was allowed to walk for 10 min. The indices measured in the apparatus were total walking distance, duration of movement, number of episode of movement, average speed of locomotion over 10 min, speed during movement, distance per movement and duration per movement.

### Statistical analysis

Values are expressed as the mean ± SE and represented graphically. Statistical significance was tested using ANOVA followed by the Bonferroni post hoc test or Student’s t-test. Correlations were calculated using the Spearman rank correlation coefficient. Statistical significance was set at p < 0.05.

## Data Availability

All data generated or analyzed during this study are included in this published article.

## Abbreviations

PA: Polyalanine
polyQ: Polyglutamine
SCA: Spinocerebellar ataxia
